# High beam quality 5 J, 200 Hz Nd:YAG laser system

**DOI:** 10.1038/lsa.2017.4

**Published:** 2017-03-24

**Authors:** Zhong-Wei Fan, Ji-Si Qiu, Zhi-Jun Kang, Yan-Zhong Chen, Wen-Qi Ge, Xiong-Xin Tang

**Affiliations:** 1 Academy of Opto-Electronics, Chinese Academy of Sciences, Beijing 100094, China; 2 National Engineering Research Center for DPSSL, Beijing 100094, China; 3 University of Chinese Academy of Sciences, Beijing 100049, China; 4 Sino-HG Applied Laser Technology Institute Company, Ltd., Tianjin 300304, China

**A high beam quality, all-solid-state Nd:YAG laser system of high-repetition frequency has been built for Thomson scattering diagnosis. A 1.7 times diffraction limited output beam at a pulse energy of 5 J at 1064 nm is achieved for the first time with a pulse duration of 6.6 ns (FWHM) and a repetition rate of 200 Hz; the output energy stability is 4.9% peak-to-valley over 6000 shots.**

## Main

**Figure 1:**
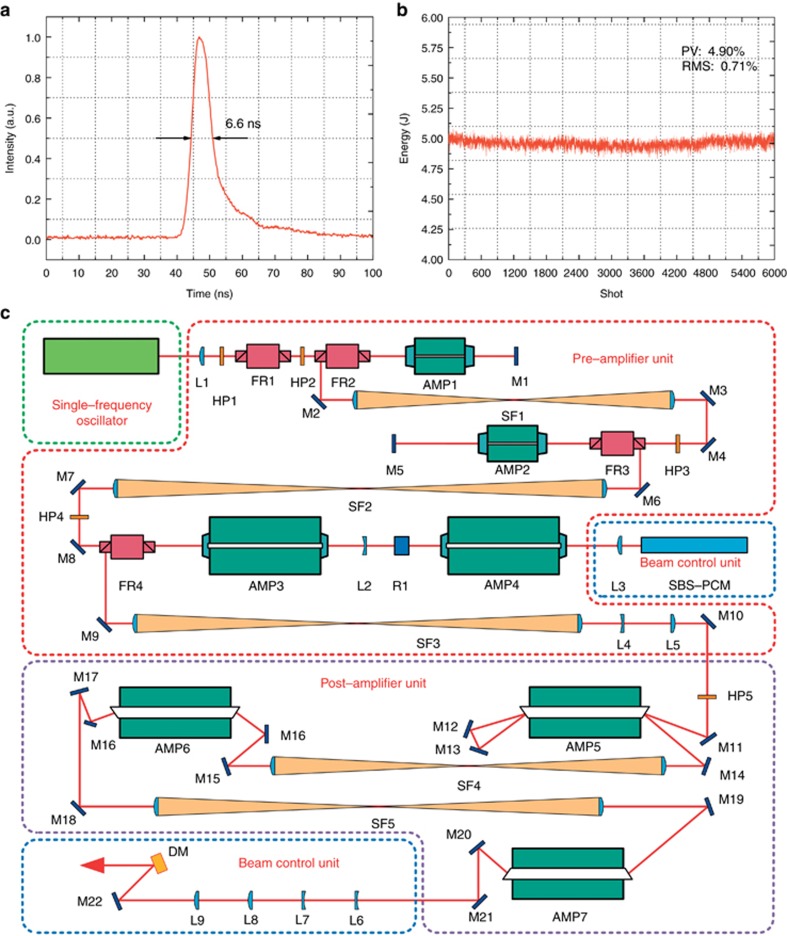
Test results of the laser pulse. (**a**) Laser pulse shape. (**b**) Curve of energy stability. (**c**) Schematic diagram of the system setup. FR, Faraday rotator; HP, half-wave plate; L, lens; M, mirror; R, 90° quartz rotator; SF, spatial filter; DM, deformable mirror.

A novel pulsed laser system (Supplementary Fig. S1) with high average power and high beam quality has recently been built by Dr Zhong-Wei Fan's group at the Academy of Opto-Electronics, Chinese Academy of Sciences. Both the laser diode side-pumped rod and slab crystals are integrated into the amplifier (AMP) system. A 1.7 times diffraction-limited output beam at a pulse energy of 5 J at 1064 nm is achieved for the first time with a pulse duration of 6.6 ns (FWHM) and a repetition rate of 200 Hz; the output energy stability is 4.9% peak-to-valley over 6000 shots. The test results are shown in Figure 1a and 1b.

The laser system is constructed in a master oscillator power amplifier (MOPA) configuration, as shown in Figure 1c, with four components: a single-frequency seed laser, pre-amplifier unit, beam control unit, and post-amplifier unit. The pre-amplifier consists of a three-stage, side-pumped rod amplifier. The dimensions of the rod crystals are φ3 mm × 67 mm with a Nd^3+^ concentration of 0.8% for AMP1 and AMP2, and φ6.35 mm × 140 mm with a Nd^3+^ concentration of 0.6% for AMP3 and AMP4. The techniques of the stimulated Brillouin scattering phase-conjugate mirror (SBS-PCM) and adaptive optics are implemented in the beam control unit to correct the wavefront distortion dynamically. The post-amplifier unit is composed of a three-stage, large slab amplifier. The dimensions of the slab crystals are 138 mm (L) × 35 mm (W) × 7 mm (D) with a Nd^3+^ concentration of 0.6% for AMP5, AMP6 and AMP7. The single-frequency seed laser produces an output power of 8.58 μJ with a pulse duration of 33.9 ns (FWHM) at a 200-Hz repetition rate. The root-mean-square (RMS) fluctuation in pulse energy is smaller than 1% and the beam quality is better than 1.12 times diffraction limited. The seed pulses first pass through the pre-amplifier and then through the control unit, at which the beam shaping is applied. The pulse energy is amplified to 300 mJ. The pulse duration is 30.5 ns and the beam quality is better than 1.4 times diffraction-limited. After passing through the post-amplifier, the pulse energy reaches 5 J with 3.2 times diffraction limited beam quality. An adaptive optics system is applied for wavefront correction, and the beam quality is improved to 1.7 times diffraction limited.

The core parts of the laser system include the single-frequency laser source, slab amplifier module with high-energy storage efficiency, and the phase-conjugated, stimulated Brillouin scattering mirror. The single-frequency source is an active Q-switching laser utilizing acousto-optic modulation. Single-longitudinal-mode operation is achieved by applying a Fabry-Pérot (FP) etalon. The length of the resonant cavity can be accurately controlled using piezoelectric ceramics, while sampling precision can be improved using a smoothing algorithm. To compensate for the influence of the environment on the length of the resonant cavity, the fuzzy proportional-integral-derivative (PID) control algorithm is implemented. With the above setup, single-frequency pulses of high stability can be achieved at the nanosecond (ns) level. The single slab amplifier can store as much as 2.5 J, with a small signal gain >5, depolarization loss <2%, pumping homogeneity better than 90%, and single-pass wavefront distortion better than 0.15 λ (RMS) when fully loaded. In the SBS-PCM, FC-770 is chosen as the SBS medium. Attributed to the specific cleaning and fine purification during the preparation processes, the load capacity of the SBS-PCM is thus improved. When pumping energy reaches 1.1 J (220 W, 200 Hz), optical breakdown is prevented and a phase-conjugate reflectivity higher than 98% is achieved.

This laser system will be adopted in the research of laser Thomson scattering diagnostics. It is expected to substantially enhance the spatial and temporal resolutions of the measurement of high-temperature plasma.

