# Supplementary material for: High beam quality 5 J, 200 Hz Nd:YAG laser system
Source: Light Sci Appl. 2017 Mar 24;6(3):e17004–. doi: 10.1038/lsa.2017.4 (PMC6062172; doi:10.1038/lsa.2017.4)
Supplement: Supplementary Figure S1 [file lsa20174x1.docx]

Supplementary Information for

High Beam Quality 5 J, 200 Hz Nd:YAG Laser System

Zhong-Wei Fan, Ji-Si Qiu, Zhi-Jun Kang, Yan-Zhong Chen, Wen-Qi Ge, Xiong-Xin Tang


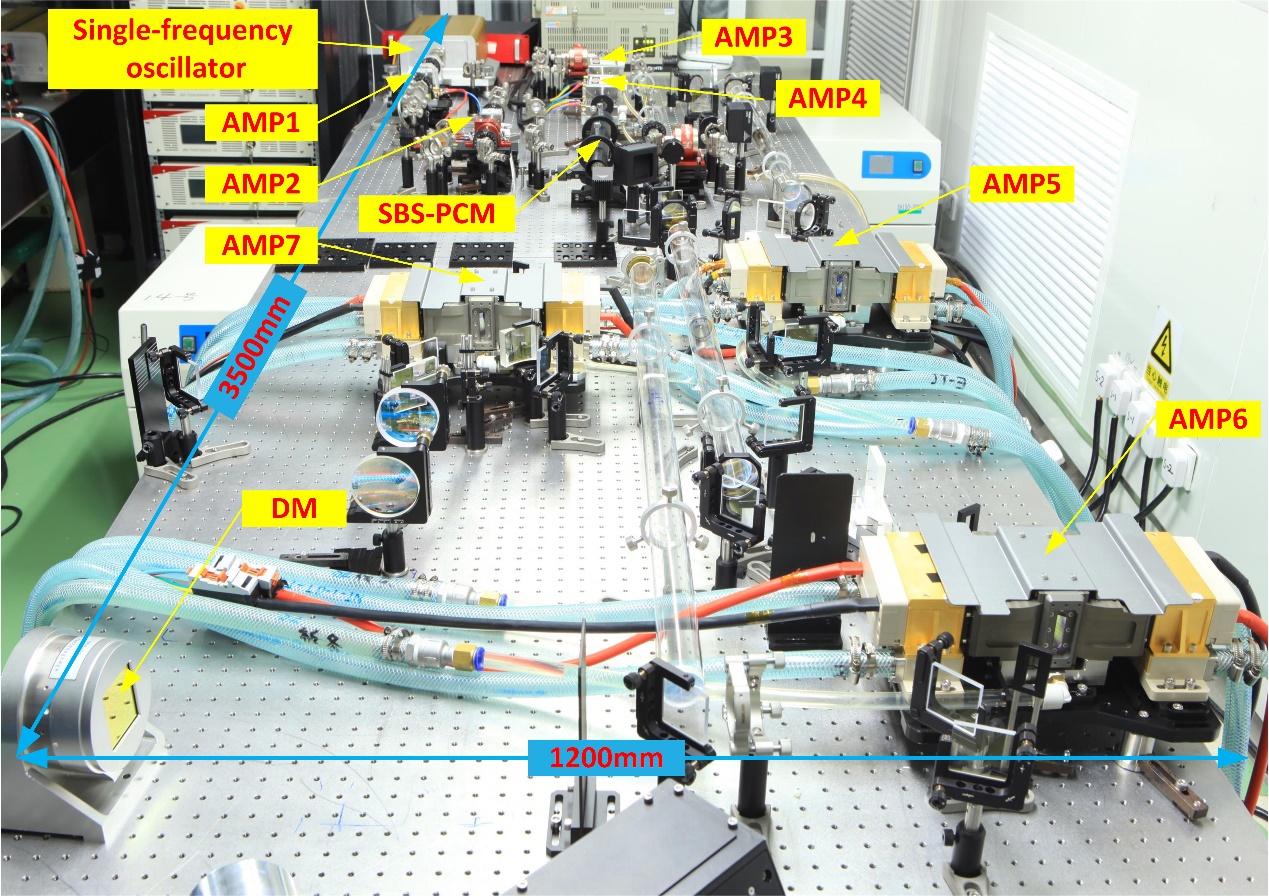


Figure S1 High beam quality 5J, 200Hz all-solid-state Nd:YAG laser system
